# *Diplazium esculentum* (Retz.) Sw. reduces BACE-1 activities and amyloid peptides accumulation in *Drosophila* models of Alzheimer’s disease

**DOI:** 10.1038/s41598-021-03142-w

**Published:** 2021-12-10

**Authors:** Thanit Kunkeaw, Uthaiwan Suttisansanee, Dunyaporn Trachootham, Jirarat Karinchai, Boonrat Chantong, Saranyapin Potikanond, Woorawee Inthachat, Pornsiri Pitchakarn, Piya Temviriyanukul

**Affiliations:** 1 Institute of Nutrition, Mahidol University, Salaya, Phuttamonthon, 73170 Nakhon Pathom Thailand; 2 Department of Biochemistry, Faculty of Medicine, Chiang Mai University, Meung, Chiang Mai, 50200 Thailand; 3 Department of Preclinical Science and Applied Animal Science, Faculty of Veterinary Science, Mahidol University, Salaya, Phuttamonthon, 73170 Nakhon Pathom Thailand; 4 Department of Pharmacology, Faculty of Medicine, Chiang Mai University, Meung, Chiang Mai, 50200 Thailand

**Keywords:** Experimental models of disease, Neurological disorders, Natural products

## Abstract

Alzheimer’s disease (AD), one type of dementia, is a complex disease affecting people globally with limited drug treatment. Thus, natural products are currently of interest as promising candidates because of their cost-effectiveness and multi-target abilities. *Diplazium esculentum* (Retz.) Sw., an edible fern, inhibited acetylcholinesterase in vitro*,* inferring that it might be a promising candidate for AD treatment by supporting cholinergic neurons. However, evidence demonstrating anti-AD properties of this edible plant via inhibiting of neurotoxic peptides production, amyloid beta (Aβ), both in vitro and in vivo is lacking. Thus, the anti-AD properties of *D. esculentum* extract both in vitro and in *Drosophila* models of Aβ-mediated toxicity were elucidated. Findings showed that an ethanolic extract exhibited high phenolics and flavonoids, contributing to antioxidant and inhibitory activities against AD-related enzymes. Notably, the extract acted as a BACE-1 blocker and reduced amyloid beta 42 (Aβ42) peptides in *Drosophila* models, resulting in improved locomotor behaviors. Information gained from this study suggested that *D. esculentum* showed potential for AD amelioration and prevention. Further investigations in vertebrates or humans are required to determine the effective doses of *D. esculentum* against AD, particularly via amyloidogenic pathway.

## Introduction

Alzheimer’s disease (AD) is an irreversible, degenerative brain disease characterized by impairment of cognitive functions, accounting for 60–80% of all dementia patients^1^. Worldwide, over 43 million people live with dementia, and this number is expected to increase dramatically, rendering AD the fifth leading cause of death^2^. Neuropathological diagnosis of an AD brain reveals the loss of cholinergic neurons from the basal forebrain to the hippocampus, resulting in reduction of choline acetyltransferase (ChAT), an enzyme responsible for the synthesis of acetylcholine (ACh), a neurotransmitter that regulates the sleep cycle, memory and learning functions^3^. Reduction of ChAT activity and ACh levels are significantly corroborated with cognitive impairment in AD cases^4^. Therefore, prevention of ACh deprivation by inhibiting acetylcholine-degrading enzymes, cholinesterases (acetylcholinesterase (AChE) and butyrylcholinesterase (BChE)) could be a therapeutic target for AD^3^. In addition to the cholinergic system, the accumulation of extracellular senile plaques consisting of deposits of amyloid beta (Aβ) peptides ultimately leads to neuronal cell death and brain atrophy^5^. Amyloid beta peptides are generated through successive cleavage of amyloid precursor proteins (APPs) by beta-secretase 1 (BACE-1) and gamma-secretase in brain tissues^6,7^. Aβ peptides as either Aβ40 or Aβ42 are thought to be secreted and aggregated as senile plaques due to their low solubility^8^, with Aβ42 the major and more neurotoxic component in amyloid plaques at the earliest stage of AD. Patients with familial AD regularly exhibit a higher ratio of Aβ42 in the brain^9^. Cleavage of APPs by BACE-1 is a rate-limiting step in Aβ production; therefore, BACE-1 inhibitors are one of the attractive targets for AD drug development^7^. Several studies on AD onset and progression have been performed; however, currently, no effective treatments are available to prevent or ameliorate this debilitating neurodegenerative disease. Drug treatments for AD are primarily designed to slow down cognitive decline and ameliorate behavioral symptoms. These drugs often induce severe side effects including nausea, vomiting and liver damage^10^. Traditional medicines from natural products are currently of interest as promising alternative candidates for the treatment of AD because of their consumption safety, cost-effectiveness and limited side effects^11^.

*Diplazium esculentum* (Retz.) Sw., a common pteridophytes in the family Athyriaceae (also known as ‘Pak-Kood’ in Thai), grows as an edible vegetable fern that is widely distributed in moist climatic areas. Only the young curly fronds are used as food ingredients in stir-fries and salads, and the fern is considered to have high economic value in Thailand. *D. esculentum* has been reported as a credible source of minerals and bioactive compounds such as phosphorus, potassium, alkaloids, flavonoids, saponins, tannins and terpenoids^12–14^ together with antibacterial, antidiabetic, antioxidant and hepatoprotective activities^15,16^. The methanolic crude extract of *D. esculentum* inhibited AChE activities in vitro, with half-maximal inhibitory concentration (IC_50_) value at 272.97 ± 19.38 μg/mL, implying that it might be a promising candidate for prevention or treatment of AD by supporting cholinergic neurons^17^. However, it is unknown whether *D. esculentum* possesses anti-AD properties in vivo, particularly in relation to the amyloid pathway. Thus, here, the anti-AD properties of *D. esculentum* extract in vitro and in *Drosophila* models of AD were elucidated. *Drosophila* models have well-defined genetic characteristics including a short life span, simplicity in genetic manipulation, and a powerful binary UAS-GAL4 transgenic system, allowing tissue-specific protein expression^18^. AD-like symptoms can be obtained from flies expressing human AD genes that resemble AD pathology presented in mice AD models^19^. Flies are also a powerful alternative model for drug screening since their anatomy exhibits an open blood vascular system, hence drugs or plant extracts can be distributed to target organs, even the brain^20^. The blood–brain barrier (BBB) is known to restrict the distribution of biomolecules (> 500 kDa), including proteins and drugs^21^. Studies in the cell models cannot overcome this problem. Thus, here, phytochemical analysis, antioxidant activities, and in vitro anti-AD properties of *D. esculentum* extract through inhibition of the key enzymes relevant to AD (AChE, BChE and BACE-1) were studied. Additionally, inhibition of BACE-1 and Aβ42 production in *Drosophila* models were also elucidated.

## Results

### Total phenolic contents (TPCs) and antioxidant activities

To determine the optimal extraction method for *D. esculentum*, the sample was extracted with gradually increased polarity index solvents including hexane, dichloromethane and ethanol. Extraction yields (%) were 10.1, 11.8 and 22.2, respectively, indicating that extraction yield increased with increasing solvent polarity. Ethanol is a suitable solvent for the extraction of various polar compounds. High recovery yield of the ethanolic extract resulted from the high concentration of polar bioactive compounds in *D. esculentum*. Likewise, lower recovery yields for dichloromethane and hexane extracts represented low contents of semi-polar and non-polar components, respectively. Bioactive compounds regarding TPCs and antioxidant activities were also tested. Table 1 shows that the ethanolic extract contained the highest TPCs compared to dichloromethane and hexane. Average TPC values of the ethanolic extract were approximately 5 times greater than dichloromethane and hexane. Average antioxidant activity of the ethanolic extract followed a similar pattern, with average DPPH radical scavenging value 12 and 30 times higher than dichloromethane and hexane, respectively. Average FRAP value of the ethanolic extract was 11 and 15 times higher than dichloromethane and hexane, respectively, while average ORAC value of the ethanolic extract was approximately 4 times higher than dichloromethane and hexane. Results implied that the sample with high TPCs extracted by ethanol also exhibited high antioxidant properties regarding DPPH radical scavenging, FRAP and ORAC assays.

**Table 1:** Total phenolic contents (TPCs) and antioxidant activities of *D. esculentum* extracted with gradually increased polarity index solvents.

Solvents	TPCs (mg GAE/g dry weight)	Antioxidant activities (μmol TE/g dry weight)		
		DPPH radical scavenging assay	FRAP assay	ORAC assay
Hexane	3.58 ± 0.25^C^	0.06 ± 0.30^B^	12.88 ± 0.29^C^	162.42 ± 6.15^B^
Dichloromethane	5.51 ± 0.44^B^	0.15 ± 0.20^B^	16.45 ± 1.52^B^	170.02 ± 9.83^B^
Ethanol	21.58 ± 0.91^A^	1.82 ± 0.54^A^	192.11 ± 10.31^A^	645.91 ± 8.74^A^

Data are presented as mean ± SD of three independent experiments. Capital letters within a column for a given parameter are significantly different at *p* < 0.05. The final concentration of the extract was 1.25 mg/mL.

### In vitro anti-Alzheimer’s disease properties

To investigate the anti-AD properties of *D. esculentum* extracted by different solvents in vitro, inhibitory enzyme assays toward AChE, BChE and BACE-1, as the major enzymes involved in AD pathogenesis, were employed. Results in Table 2 show that all *D. esculentum* extracts displayed anti-AChE, anti-BChE and anti-BACE-1 activities with varying degrees of inhibition. The ethanolic fraction exhibited the highest anti-AChE activity (46.15 ± 6.17% inhibition). Ethanol was the best solvent for obtaining bioactive compounds against AChE at approximately 2 to 5 times greater than dichloromethane and hexane. Conversely, Table 2 also revealed that anti-BChE activity of all *D. esculentum* extracts was more pronounced than anti-AChE activities. However, statistical analysis indicated that all fractions possessed the same inhibitory activities against BChE.

**Table 2:** Percentage enzyme inhibitory activities of *D. esculentum* extracted with hexane, dichloromethane and ethanol against AChE, BChE and BACE-1.

Solvents	Enzyme inhibitory activities (%)		
	AChE	BChE	BACE-1
Hexane	15.59 ± 3.23^C^	47.43 ± 4.11^A^	69.76 ± 4.16^A^
Dichloromethane	24.19 ± 3.92^B^	49.85 ± 4.29^A^	68.54 ± 0.44^A^
Ethanol	46.15 ± 6.17^A^	53.12 ± 5.80^A^	55.91 ± 5.32^B^

Data are presented as mean ± SD of three independent experiments. Capital letters within a column for a given parameter are significantly different at *p* < 0.05. The final concentration of the extract was 1.25 mg/mL.

*D. esculentum* extracts were tested for their inhibitory properties against BACE-1, an enzyme involved in the amyloidogenic pathway. Inhibition of BACE-1 is currently one of the AD drug targets. All fractions appeared to inhibit BACE-1 with high efficacy (above IC_50_ value). Hexane and dichloromethane fractions exhibited anti-BACE-1 activities significantly higher than the ethanolic fraction (Table 2). This result was interesting since the ethanolic fraction of *D. esculentum* constantly demonstrated high TPC, antioxidant activity, anti-AChE and anti-BChE activities (Tables 1, 2). Bioactive compounds inhibiting BACE-1 may be different from those inhibiting AChE and BChE. Since hexane and dichloromethane extracts showed high anti-BACE-1 activities, the active compounds obstructing BACE-1 were probably hydrophobic, non-polar compounds.

Extraction yield and in vitro data regarding TPCs, antioxidant and enzyme inhibitory activities revealed that the ethanolic fraction of *D. esculentum* was the most potent among all the tested extracts (Tables 1, 2). Thus, only the ethanolic fraction of *D. esculentum* was subjected to phenolic compound analysis utilizing high-performance liquid chromatography (HPLC) (Supplementary Figure S1 and S2). Only two flavonoids were identified, including quercetin (1463.69 ± 29.07 μg/g extract), and its glycosylated form, rutin (760.77 ± 26.93 μg/g extract) (Table 3).

**Table 3:** Quantification of rutin and quercetin in the ethanolic extract of *D. esculentum*.

	Flavonoids (µg/g extract)	
	Rutin	Quercetin
Ethanolic extract of *D. esculentum*	760.77 ± 26.93	1,463.69 ± 29.07

### Suppression of BACE-1 activities and Aβ42 accumulation in flies co-expressing APP-BACE-1

**Figure 1:**
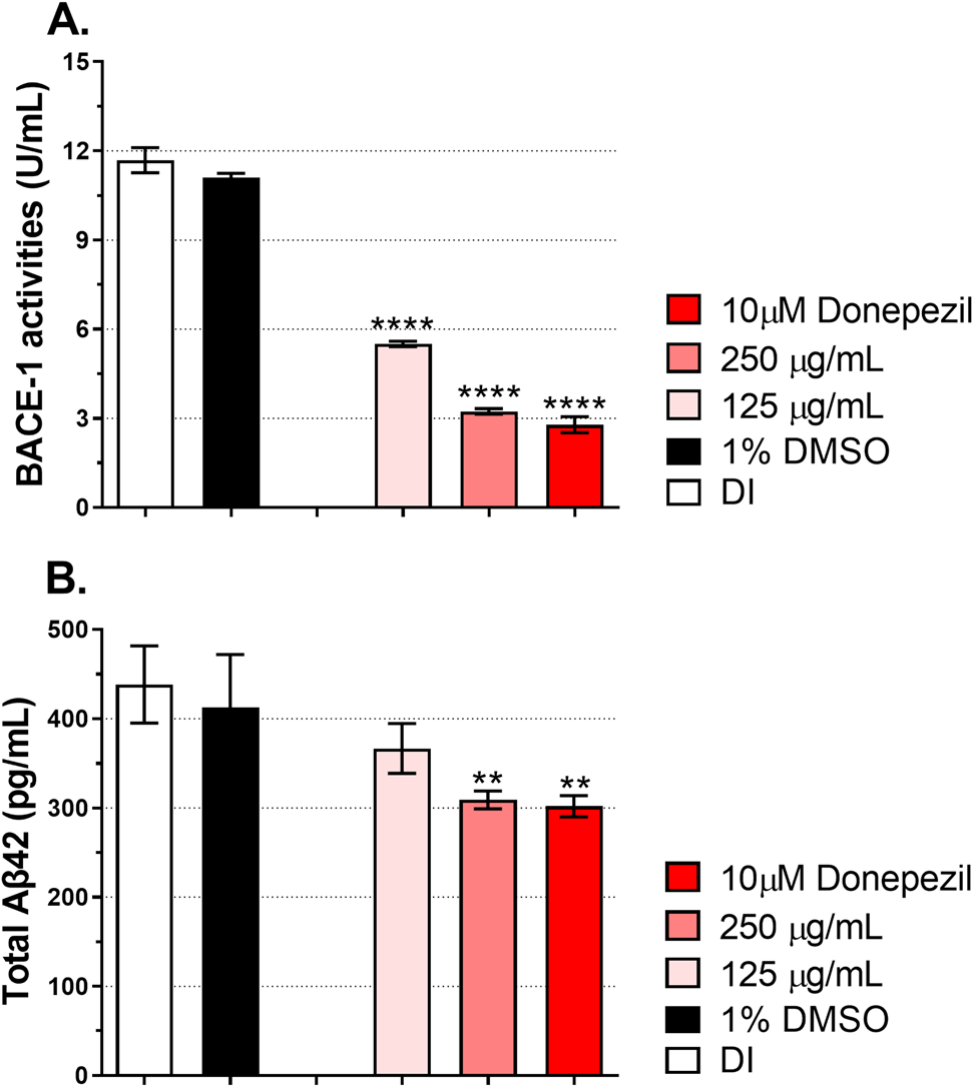
Effect of the ethanolic extract of *D. esculentum* on (**A**) BACE-1 activities and (**B**) Aβ42 accumulation in the *Drosophila* models of AD. AD-free flies (elav-Gal4) were cultured on a normal medium, and AD flies (APP-BACE-1) were cultured on a medium containing deionized water (DI), 1% DMSO, 125 µg/mL and 250 µg/mL of *D. esculentum* and 10 µM donepezil for 28 days before assay for either BACE-1 activities or Aβ42 levels. Values are mean ± SD of three assays and statistical significance was analyzed against AD flies (DI) by one-way ANOVA followed by Tukey’s test. **, *p* < 0.01) and ****, *p* < 0.0001.

The ethanolic extract of *D. esculentum* inhibited BACE-1 activity by approximately 50% in vitro (Table 2). Hence, the effect of *D. esculentum* extracts on BACE-1 activity in vivo using *Drosophila* was further determined. To represent human amyloidogenic pathway, flies carrying human amyloid precursor proteins (APPs) and human beta-secretase 1 (BACE-1) (APP-BACE-1) were utilized. Virgin females of the pan-neuronal elav-Gal4 driver were crossed with UAS-APP-BACE-1 males, resulting in F1 flies that co-expressed human APPs and BACE-1 (called AD flies). Moreover, elav-Gal4 flies were also included as AD-free flies. Deionized water (DI) was used as a chemical-free control, and 1% DMSO was used as a solvent control to dissolve donepezil and the extract. After eclosion, flies were treated with DI, 1% DMSO, 125 and 250 µg/mL of *D. esculentum* as well as 10 µM donepezil for 28 days. Afterward, head lysates were prepared without protease inhibition and subjected to BACE-1 assay. Figure 1A shows that both AD flies treated with DI or 1% DMSO displayed the same levels of BACE-1 function, indicating that DI and 1% DMSO did not affect BACE-1 activity in vivo, while AD-free flies had no detectable BACE-1 activity as expected. BACE-1 activity decreased by two folds in AD flies receiving 125 µg/mL of *D. esculentum*. This effect was considerably enhanced when AD flies received 250 µg/mL of *D. esculentum* and 10 µM donepezil. Donepezil was reported to inhibit BACE-1 enzymes at an IC_50_ of 1.5 µM^22^. Results suggested that the ethanolic fraction of *D. esculentum* ingested by flies inhibited BACE-1 activity in a dose-dependent manner.

Proteolytic cleavage of APPs by BACE-1 resulted in the formation of amyloid beta 42 (Aβ42) peptides, a hallmark of AD^23^. Suppression of BACE-1 activity presented in AD flies raised the question whether the ethanolic extract of *D. esculentum* could prevent Aβ42 accumulation in AD flies. On day 28 of treatment, fly heads were collected and lysed with a lysis buffer containing protease and phosphatase inhibitors to prevent amyloid degradation. Equal amounts of protein supernatants were used to quantify human Aβ42 using the ELISA method. In agreement with previous studies, Fig. 1B shows that AD flies treated with DI or 1% DMSO accumulated high Aβ42 at similar levels, while Aβ42 was not detected in elav-Gal4 (AD-free flies, data not shown). Results indicated that the fly model was representative of human amyloidogenic pathway. AD flies that received 250 µg/mL of *D. esculentum* as well as 10 µM donepezil exhibited significantly decreased levels of Aβ42 compared to DI and 1% DMSO. *D. esculentum* and donepezil influenced BACE-1 activity (Fig. 1B). However, this was not potent enough to reduce Aβ42 deposits, although a low concentration of *D. esculentum* at 125 µg/mL reduced BACE-1 activity by approximately 50% in the tested flies. The ethanolic fraction of *D. esculentum* may prevent AD by reducing Aβ42 deposits via BACE-1 inhibition in a dose-dependent manner.

### Climbing behavior in flies co-expressing APP-BACE-1

**Figure 2:**
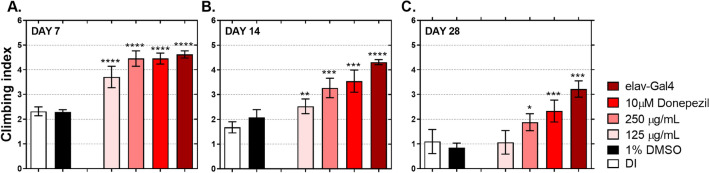
Effects of the ethanolic extract of *D. esculentum* on *Drosophila* climbing ability. AD-free flies (elav-Gal4) cultured on a normal medium, and AD flies (APP-BACE-1) cultured on a medium containing deionized water (DI), 1% DMSO, 125 µg/mL and 250 µg/mL of *D. esculentum* and 10 µM donepezil for the indicated days were assessed for climbing ability. Values are mean ± SD of three assays and statistical significance was analyzed against AD flies (DI) by one-way ANOVA followed by Tukey’s test. *, *p* < 0.05; **, *p* < 0.01; ***, *p* < 0.001 and ****, *p* < 0.0001.

Results in Fig. 1A and B showed that the ethanolic extract of *D. esculentum* inhibited BACE-1 activity in flies expressing APP and BACE-1, resulting in reduced production of Aβ42 in fly brains. Uniquely, most neurodegenerative diseases, including AD, can be characterized by age-dependent deterioration in climbing behavior in *Drosophila*. Thus, reduction in Aβ42 accumulation leading to improvement of *Drosophila* climbing behavior was examined. As illustrated in Fig. 2A, the AD-free flies (elav-Gal4) climbed at a climbing index of 4.5 on day 7 after eclosion. The climbing index gradually reduced with increased fly age (Fig. 2B,C), supporting the age-dependent phenotype. In AD flies, both those with DI or 1% DMSO showed severe climbing index as early as day 7. This defect increased with aging, confirming that AD caused severe damage to the fly brain, leading to poor climbing ability. Interestingly, flies with two different concentrations of *D. esculentum* (125 µg/mL and 250 µg/mL) and 10 µM donepezil showed reduced climbing defects. On day 7 after eclosion, compared to AD and AD-free control flies, flies receiving 250 µg/mL of *D. esculentum* and 10 µM donepezil could fly as high as the AD-free flies, while flies that received 125 µg/mL of *D. esculentum* showed moderate rescuing ability. Although flies that received 250 µg/mL of *D. esculentum* climbed progressively slower as they aged, the climbing index was statistically similar to flies with 10 µM donepezil at all times tested, suggesting the rescuing activity of *D. esculentum* at 250 µg/mL in climbing behavior. Unfortunately, *D. esculentum* at 125 µg/mL delayed the climbing index by up to 14 days; therefore, this dose of ethanolic extract of *D. esculentum* should not be used, even though it can suppress Aβ42 accumulation (Fig. 1B).

### Aβ42 accumulation and climbing behavior in flies expressing Aβ42

**Figure 3:**
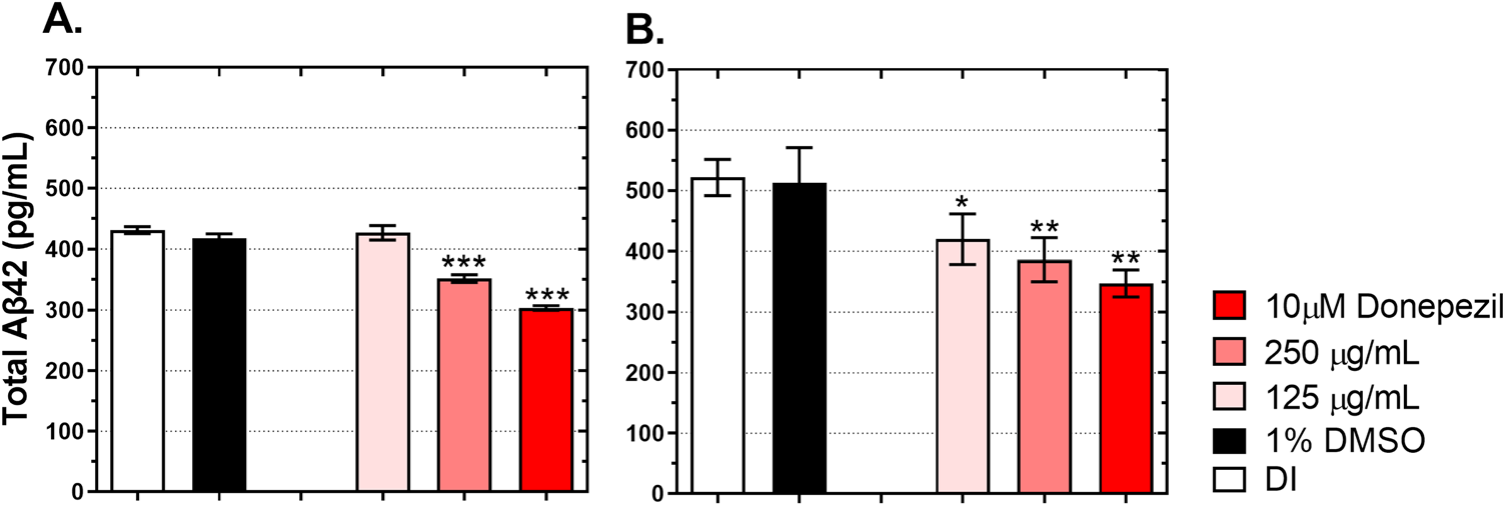
Effect of the ethanolic extract of *D. esculentum* on Aβ42 accumulation in *Drosophila* models as (**A**) flies expressing Aβ42 (chr.2) and (**B**) flies expressing Aβ42 (chr.3). AD-free flies (elav-Gal4) were cultured on a normal medium, while AD flies (Aβ42) were cultured on a medium containing deionized water (DI), 1% DMSO, 125 µg/mL and 250 µg/mL of *D. esculentum* and 10 µM donepezil for 28 days and then assayed for Aβ42 levels. Values are mean ± SD of three assays and statistical significance was analyzed against AD flies (DI) by one-way ANOVA followed by Tukey’s test. *, *p* < 0.05; **, *p* < 0.01; ***, *p* < 0.001.

Figure 1 shows that *D. esculentum* extract reduced Aβ42 quantity by influencing BACE-1 activities. To determine whether *D. esculentum* reduced Aβ42 deposit, two strains of flies carrying Aβ42 comprising of Aβ42 chr.2 and Aβ42 chr.3 were investigated. Virgin females of elav-Gal4 were crossed with either UAS-Aβ42 chr.2 or UAS-Aβ42 chr.3 males, resulting in F1 flies that expressed human Aβ42 peptides (called AD flies). Elav-Gal4 flies were also included as AD-free flies. After eclosion, the AD flies were subsequently treated with DI, 1% DMSO, 125 and 250 µg/mL of *D. esculentum* as well as 10 µM donepezil. Flies were treated for 28 days. On day 28, fly heads were collected and quantified for human Aβ42, as previously described. Figure 3A and B show that AD flies treated with DI or 1% DMSO had high levels of Aβ42 peptide, whereas elav-Gal4 did not express Aβ42 (data not shown). AD flies that received 250 µg/mL of *D. esculentum* as well as 10 µM donepezil showed significantly decreasing Aβ42 peptides compared to DI and 1% DMSO. However, *D. esculentum* at 125 µg/mL did not prevent Aβ42 deposit when Aβ42 chr.2 flies were used. By contrast, *D. esculentum* at 125 µg/mL exhibited considerable Aβ42 suppression when Aβ42 chr.3 flies were used (Fig. 3B). It was not possible to explain this dissimilarity between Aβ42 chr.2 and Aβ42 chr.3; however, *D. esculentum* showed neuroprotective properties by suppression of Aβ42 deposit.

**Figure 4:**
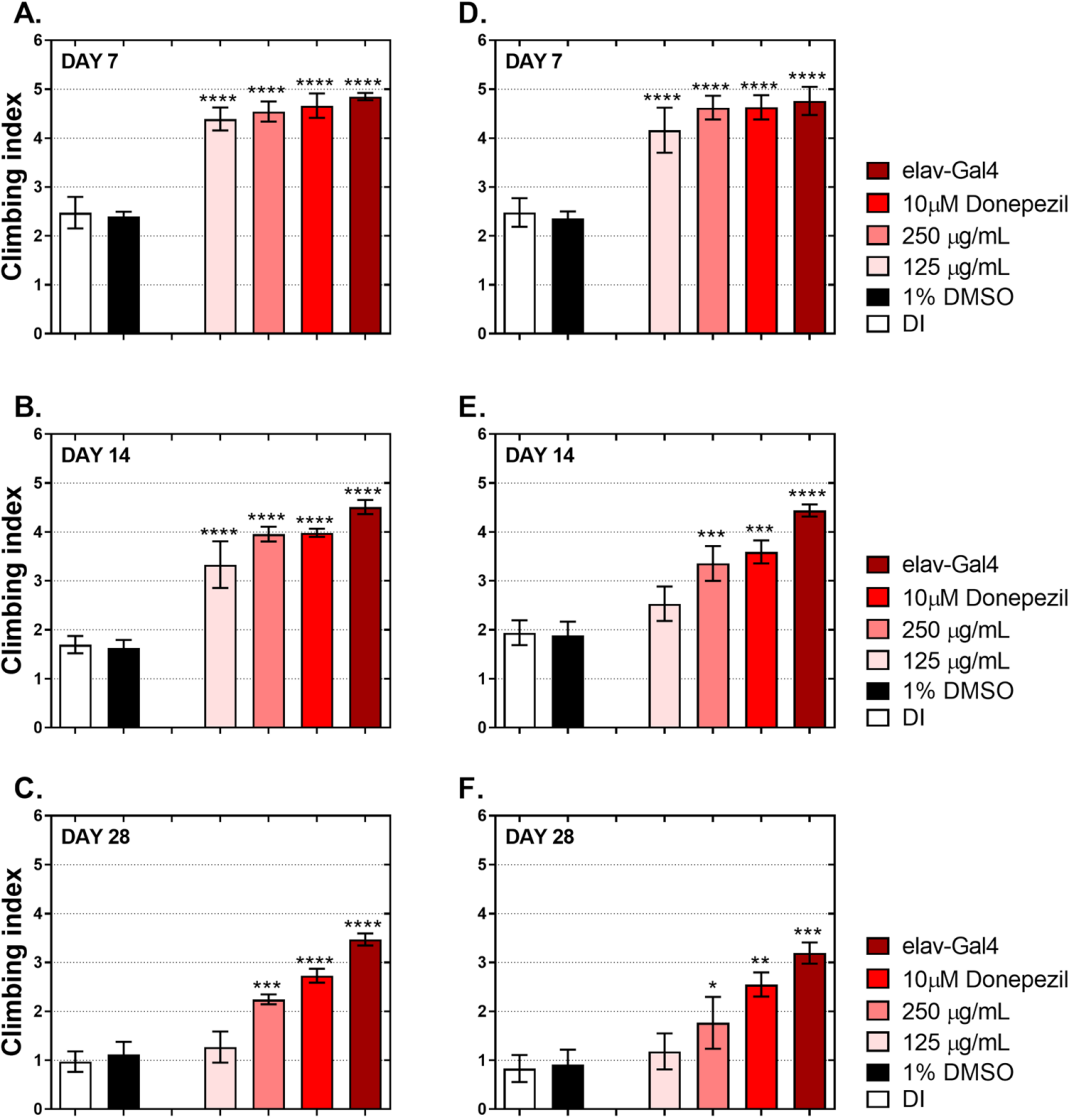
Effect of the ethanolic extract of *D. esculentum* on climbing ability of flies expressing Aβ42, (**A**–**C**) flies expressing Aβ42 (chr.2), and (**D**–**F**) flies expressing Aβ42 (chr.3). AD-free flies (elav-Gal4) were cultured on a normal medium, while AD flies (Aβ42) were cultured on a medium containing deionized water (DI), 1% DMSO, 125 µg/mL and 250 µg/mL of *D. esculentum* and 10 µM donepezil for the indicated days and then assayed for climbing ability. Values are mean ± SD of three assays and statistical significance was analyzed against AD flies (DI) by one-way ANOVA followed by Tukey’s test. *, *p* < 0.05; **, *p* < 0.01; ***, *p* < 0.001.

As shown in Fig. 3, the ethanolic extract of *D. esculentum* reduced Aβ42 accumulation in both strains of flies expressing Aβ42. *D. esculentum* reduced Aβ42 aggregation while enhancing Aβ42 clearance from fly brains. Reduction in Aβ42 accumulation and whether it led to improvement of *Drosophila* behaviors was tested. As demonstrated in Fig. 4, AD-free flies (elav-Gal4) had a climbing index of 5 on day 7 after eclosion. The climbing index declined slowly on day 14 and day 28 (Fig. 4). AD flies (Aβ42 chr.2) fed with DI or 1% DMSO as control were approximately two-folds slower compared to AD-free flies, demonstrating severe climbing deficits as early as day 7. Also, this defect continuously influenced climbing ability at day 14 and day 28, confirming that Aβ42 peptides caused severe neurotoxicity, eventually leading to poor climbing ability (Fig. 4A–C). During treatment, flies with two different concentrations of *D. esculentum* (125 µg/mL and 250 µg/mL) and 10 µM donepezil showed significant climbing improvement compared to AD flies. However, flies that received 125 µg/mL of *D. esculentum* showed moderate rescuing ability that lasted for 14 days. AD flies treated with 250 µg/mL of *D. esculentum* exhibited comparable rescuing ability with flies treated with 10 µM donepezil, albeit to a lesser extent. AD flies (Aβ42 chr.3) were used and tested following the same procedure as Aβ42 chr.2 (Fig. 4D–F). Results concurred when Aβ42 chr.2 was used, indicating that different locations of gene modification did not interfere with AD pathogenesis in the AD flies. Importantly, this suggested that the ethanolic fraction of *D. esculentum* at 250 µg/mL could be used as a therapeutic dose in AD flies when taken together with the obtained data.

## Discussion

Alzheimer’s disease (AD) is an irreversible, degenerative brain disease leading to dementia. Regrettably, current medicines show side effects, low efficacy, and cannot rescue AD pathogenesis. AD complexities are associated with several pathways involving oxidative stress, tau hyperphosphorylation, inflammation, cholinesterases, and BACE-1 functions; thus, safe and multi-targeted medicine could be ideal for AD drugs. Vegetables and plant extracts are currently under-investigated for their health-promoting activities, including anti-AD properties. Several pieces of evidence illustrate the potential anti-AD properties of phytochemicals, including phenolic acids and flavonoids both in vitro and in vivo^24^. Here, results elucidated the anti-AD properties of an edible fern with high economic value as *D. esculentum *in vitro and using *Drosophila* models of AD. The main findings were (1) ethanol was an appropriate solvent for extracting anti-AD compounds from *D. esculentum*, (2) ethanolic extracts were rich in phenolic compounds, particularly rutin and its aglycone, quercetin, that might contribute to antioxidant and inhibitory activities against AD-related enzymes, and (3) the ethanolic extract inhibited BACE-1 activities, a rate-limiting step in amyloid beta production, and also directly interfered with Aβ42 levels in *Drosophila* brain.

Based on the high antioxidant activities detected in hydrophilic solvents (ethanol), the antioxidative agents presented in *D. esculentum* might be flavonoids and phenolic acids that dissolve better in hydrophilic solvents compared to hexane and dichloromethane. Ethanol is a versatile solvent that is preferably used to dissolve polar substances with some contamination of non-polar compounds. Phenolics such as flavonoids are plant secondary metabolites reported for their health benefits, including their high antioxidant properties^25^. Results indicated that the ethanolic fraction from *D. esculentum* possessed high TPCs and antioxidant activities and showed correlation between the amount of TPCs and antioxidant properties (Table 1). This finding was consistent with a previous study demonstrating that the methanolic fraction of *D. esculentum* had high antioxidant properties compared to the chloroform fraction^26^. An HPLC analysis of the ethanolic fraction of *D. esculentum* found only rutin and quercetin. Interestingly, an earlier report showed that *D. esculentum* is rich in procyanidin, quercetin-3-rutinoside (rutin), kaempferol-3-rutinoside, quercetin-3-glucoside, and eriodictyol 5-O-methyl ether 7-β-D-xylosylgalactoside, which are flavonoids^27^. Flavonoid structures mainly consist of hydroxyl groups, 2–3 double bonds, and 4-oxo functions, promoting antioxidant activities^28^.

Results revealed that the ethanolic fraction exhibited high enzyme inhibitory activities toward AChE, BChE and BACE-1 covering both cholinergic and amyloid hypotheses at 46, 53 and 56%, respectively (Table 2). Findings implied that the AChE, BChE and BACE-1 inhibitors present in *D. esculentum* might be polar compounds, in parallel with antioxidant data. Two flavonoids detected in *D. esculentum* extract, rutin and quercetin, might be responsible for these inhibitory activities. However, it has been documented that rutin and quercetin inhibited AChE reaction with half maximal inhibitory concentration (IC_50_) of 219 and 181 µM, respectively, while these two flavonoids also inhibited BChE reaction with IC_50_ values of 288 and 203 µM, respectively^29^. Nevertheless, the concentration of rutin and quercetin presented in the *D. esculentum* extract during enzyme assays were 1.56 and 6.05 µM, respectively, implying that rutin and quercetin may synergist with other unknown active compound(s) in the extract to inhibit AChE and BChE activities, which should be further explored. Interestingly, quercetin effectively inhibited BACE-1 reaction with IC_50_ values of 5.4 µM^30^. In addition, among 448 clinical compounds (NIH clinical collection), rutin was identified as a potential BACE-1 inhibitor (effective at a dose of 1 μM) by a systematic approach called AβPP-selective BACE inhibitors (ASBI)^31^. Thus, due to their low IC_50_ values against BACE-1, these flavonoids may act as strong inhibitors against BACE-1, leading to high enzyme inhibition detected in the assay containing *D. esculentum* ethanolic extract (Table 2).

We molecularly explored our finding in-depth in vivo using *Drosophil*a models. Over the last decade, an increasing number of studies have used *Drosophila* as a model to study AD. Our model was developed based on Wang et al*.*^32^, who assessed the anti-AD effect of curcuminoids in flies co-expressing human APPs and BACE-1. Their data revealed that curcuminoids could rescue rough eye phenotypes, and climbing behaviors via BACE-1 inhibitory activities, confirming the appropriate use of *Drosophila* as a model for studying plant extracts on amyloidogenic pathway. Two hypotheses were tested. The first was the effect of *D. esculentum* extract on *Drosophila* co-expressing human APPs and BACE-1 that represent human amyloid cascades. The second was the direct effect of the extract on *Drosophila* expressing human Aβ42 peptides. This model was assessed to determine the direct role of *D. esculentum* on prevention of Aβ42 toxicity. We found that the ethanolic extract acted as a BACE-1 blocker and also directly reduced Aβ42 peptides in vivo (Figs. 1, 2, 3 and 4). Although a lower concentration of *D. esculentum* might be used in the early stage of AD, a higher concentration is recommended for progressive deterioration of AD. The *D. esculentum* extract inhibited BACE-1 activity in fly brains. This infers that the extract can be diffused across the *Drosophila* blood–brain barrier (BBB). One of the major obstacles in AD drug development is the inability to traverse the BBB. A compound that can pass the BBB should comprise lipophilic molecules with molecular weight less than 400–500 kDa^21^. Phenolic compounds may be suitable as AD drug candidates due to their small molecular weight (< 400 Da). There are two mechanisms underlying inhibition of BACE-1 as (1) direct interaction between compounds and enzymes, and (2) interference of BACE-1 expression. The first was demonstrated by Shimmyo et al*.* They found that quercetin inhibited BACE-1 activities with IC_50_ values of 5.4 µM via direct inhibition at the enzyme active site^30^. For the second mechanism, Huang et al. showed that BACE-1 expression could be suppressed via estrogen receptor β (ERβ) and nuclear factor-kappa B (NFκB) signaling by curcumin, a polyphenol from turmeric^33^. The orthologs of these proteins can also be found in *Drosophila*^34,35^. Thus, *D. esculentum* may directly inhibit BACE-1 activities, BACE-1 expression and eventually Aβ42 formation. We predict that quercetin, rather than rutin, was an active component in *D. esculentum* extract because rutin will be hydrolyzed to quercetin in the digestive system, allowing quercetin to be absorbed^36^.

How *D. esculentum* directly reduces Aβ42 peptides in *Drosophila* remains unclear (Fig. 4). This study found that *D. esculentum* contains phenolic compounds such as quercetin (Table 3). These compounds might directly prevent Aβ40 and Aβ42 formation and extension^37^. Quercetin has been reported to reduce Aβ42 toxicity in *Drosophila*-expressing Aβ42 peptides via restoring gene expression disturbed by Aβ toxicity, including cyclin B^38^. Furthermore, it has been reported that cholinesterases (AChE and BChE) are associated with aggregation of Aβ peptides and acetylcholinesterase inhibitor (donepezil) reduced acetylcholinesterase-mediated Aβ aggregation^39^. Thus, it could be possible that donepezil and the *D. esculentum* extract, which exhibited AChE and BChE inhibitory activities (Table 2) reduced Aβ42 accumulation in flies expressing Aβ42 via inhibition of AChE and BChE. Accumulation of amyloid peptides in nerve cells formed senile plaques and generated reactive oxygen species (ROS)^40^. ROS are derived from degenerating neurons. Our results showed that *D. esculentum* also contains unidentified phenolic acids and flavonoids that might contribute to high antioxidant activities, particularly through hydrogen atom transfer (HAT), as indicated by the ORAC data (Table 1)^41^. Hence, the role of antioxidant activity of *D. esculentum* cannot be excluded.

Although, in the present study, there is no evidence that the decrease in Aβ is a direct cause of the increase in climbing ability, several articles have shown that amyloid peptide expression in the *Drosophila* lead to (1) apoptotic cell death in the fly brain^42^, (2) defect in fly neuroanatomy^43^, (3) amyloid peptide deposit and aggregate in fly central nervous system (CNS)^43–45^, (4) cell body and neuropil degeneration^45^ and (5) reduced glial cell number in Aβ-expressing brains^46^. All defects (1–5) lead to poor climbing ability. Hence, our flies which expressed Aβ could also mimic the same finding. However, further experiments, including apoptosis in fly brain tissues, neurodegeneration in the fly brain, Aβ-mediated ROS levels and degree of Aβ aggregation should be considered.

## Material and methods

### Sample preparation and extraction

*Diplazium esculentum* (Retz.) Sw. (*D. esculentum*) was collected from Chiang Mai, Thailand. The sample collection was conducted following the guidelines and regulations of the legislation and the sample was identified by Dr. Kanchana Pruesapan (taxonomist), Plant Varieties Protection Division, Department of Agriculture, Bangkok. The plant sample was deposited in Bangkok Herbarium (BK), Bangkok, Thailand. The herbarium voucher specimen is BK069943. Edible part of *D. esculentum* (young leaves and young stems) was washed with deionized water and cut into small pieces. The sample was freeze-dried (Heto PowerDry PL9000, Heto Lab Equipment, Allerød, Denmark) and then ground into fine powder. Moisture content of the powder was determined using a Halogen moisture analyzer (HE53 series, Mettler-Toledo AG, Greifensee, Switzerland). The dry powder was extracted with hexane or dichloromethane or ethanol at 30 °C for 2 h. The mixture was then centrifuged at 3000 g for 20 min and the supernatant was subsequently collected. The solvent was removed using a rotary evaporator (model Eyela N-1200 Series). The dry extract was weighed, redissolved in DMSO, and kept at − 20 °C until required for further analysis.

### Phytochemical analysis

Total phenolic contents (TPCs) were determined according to the Folin-Ciocalteu method adapted from Ainsworth and Gillespie^47,48^. The TPCs were measured at a wavelength of 765 nm using a microplate reader. Gallic acid (10, 20, 40, 60, 80, 100 and 200 μg/mL) was used as the standard, and TPCs were expressed in gallic acid equivalents (GAE) per 1 g dry weight of sample.

The ethanolic fraction of *D. esculentum* was investigated for phenolic compounds using high-performance liquid chromatography (HPLC) (UtiMate HPLC with a HPG-3400SD pump equipped with photodiode array detector from Dionex, Sunnyvale, CA, USA) and C18 column (250 × 4.6 mm, 5 µm) (Agilent Technologies, Santa Clara, CA, USA), following sample preparation and HPLC conditions previously described^49^. The chromatogram was compared to phenolic standards, including apigenin, caffeic acid, catechin, chlorogenic acid, ferulic acid, gallic acid, hesperitin, isorhamnetin, kaempferol, luteolin, myricetin, naringenin, quercetin, rosmarinic acid and vanillic acid. In addition, 10 mg/mL of extract was diluted in 1 mL of methanol and injected onto the column, followed by the aforementioned HPLC condition, to measure the glycosylated form of quercetin, quercetin-3-rutinoside or rutin.

### Antioxidant activities

The established antioxidant assays including 2,2-diphenyl-1-picrylhydrazyl (DPPH) scavenging activity, ferric ion reducing antioxidant power (FRAP), and oxygen radical absorbance capacity (ORAC)^50–53^ were employed to evaluate the antioxidant activities of hexane, dichloromethane and ethanolic fraction of *D. esculentum*.

### Inhibitory activities against AD-related enzymes

AChE inhibitory activities were assayed as formerly detailed by Jung et al. 2009 and Temviriyanukul et al. 2020^53–55^. In brief, 20 ng of *Electrophorus electricus* AChE (1,000 units/mg, 100 μL) in 50 mM KPB (pH 7.0) was mixed with 16 mM 5,5-dithio-bis-(2-nitrobenzoic acid) (DTNB, 10 μL), 0.8 mM acetylthiocholine (40 μL) in 50 mM KPB (pH 7.0), and *D. esculentum* extract (50 μL). The reaction was measured using a microplate reader (Synergy™ HT 96-well UV–visible spectrophotometer with Gen5 data analysis software from BioTek Instruments, Inc., Winooski, VT, USA) at 412 nm. Inhibitory percentage was calculated as follows:

$$\% \;{\text{inhibition}} = \left( {1 - \frac{B - b}{{A - a}}} \right) \times 100,$$

where *A* is the initial velocity of the reaction with enzyme, *a* is the initial velocity of the reaction without enzyme, *B* is the initial velocity of the enzyme reaction with extract, and *b* is the initial velocity of the reaction with extract but without enzyme.

The BChE inhibitory activities of *D. esculentum* extract were tested as AChE assay. A total of 100 ng equine serum BChE (≥ 10 units/mg protein, 100 μL) in 50 mM KPB (pH 7.0) containing 1 mM MgCl_2_ and 0.1 mM butyrylthiocholine (BTCh) in 50 mM KPB (pH 7.0) were used as the reaction enzyme and substrate, respectively^54,55^.

Fluorescence resonance energy transfer (FRET) on a BACE-1 activity detection kit (Sigma-Aldrich, MO, USA) was used to determine BACE-1 activity following the manufacturer’s instructions.

### *Drosophila* stocks and ethical approval

The elav-GAL4 (w[*]; P{w[+mC] = GAL4-elav.L}CG16779[3]) (BDSM 8760), UAS-APP-BACE-1 (w[1118]; P{w[+mC] = UAS-APP695-N-myc}TW6, P{w[+mC] = UAS-BACE1.Exel}1b) (BDSM 33798), UAS-Aβ42 (chr.2) (w[1118]; P{w[+mC] = UAS-APP.Abeta42.B}m26a) (BDSM 33769) and UAS-Aβ42 (chr.3) (w[*]; P{w[+mC] = UAS-Abeta1-42.G}3) (BDSM 32037) were obtained from Bloomington *Drosophila* stock center (Bloomington, IN, USA). All fly stocks were maintained in a standard medium at 25 °C. Based on the UAS/GAL4 system, crossing between an *elav-GAL4* (driver) and a UAS strain resulted in F1 progenies expressing the desired expression of a specific protein in the fly brain. Schematic diagrams of *Drosophila* crosses and studies are illustrated in supplementary Figure S3. The *Drosophila* studies were approved by Mahidol University Institute of Animal Care and Use Committee (MU-IACUC) (COA.No.MU-IACUC 2017/024) and the Institute of Nutrition, Mahidol University Institutional Animal Care and Use Committee (INMU-IACUC) (COA.No.MU-IACUC 2021/01).

### *Drosophila* treatment

The newly eclosed F1 progenies (1–2 days old) were fed with instant *Drosophila* medium blue (Carolina Biological Supply Company, Burlington, North Carolina, USA) containing safety doses of *D. esculentum* ethanolic fraction (125 and 250 µg/mL), 1% DMSO or 10 µM donepezil (cholinesterase and BACE-1 inhibitor^22^) and cultured at 28 °C for the indicated time.

### Climbing assay

To determine the neurodegenerative defect underlining locomotory coordination, the climbing or negative geotaxis assay was used^56^. In brief, on the indicated day after treatment, flies were transferred to a clean vial without anesthesia and given 15 min for acclimatization at 25 °C. The climbing index of each tested experiment was determined as follows: the number of flies in each score multiplied by the score they reached, and then divided by the total number of flies in each group.

### Determination of Aβ42 Levels and BACE-1 activities in fly brain lysate

For Aβ42 quantification, twenty-five to thirty fly heads were separated and collected using centrifugation and then mechanically homogenized in 100 µL of 5 M guanidine-HCl containing Protease Inhibitor Cocktail (Thermo Scientific™). The samples were then centrifuged at 12,000* g* for 15 min at 4 °C^57,58^. Protein supernatants were determined for protein concentration using Pierce BCA Protein Assay Kit (Bicinchoninic acid assay, Thermo Scientific™). Before sample loading, serial dilution of supernatants was performed using ELISA diluent buffer containing Protease Inhibitor Cocktail following the manufacturer’s instructions (Life Technologies, Invitrogen). The samples were then measured at 450 nm. Concentration of Aβ42 peptides was calculated and compared with the standard control (recombinant human Aβ42). Data are presented as mean ± SD of three experiments.

For BACE-1 activities, twenty-five to thirty fly heads were separated and collected using centrifugation and then mechanically homogenized in 100 µL of T-PER™ Tissue Protein Extraction Reagent. Then, 2 μL of protein samples (conc. 3 mg/mL) were used to measure BACE-1 activity in the brain lysate. All reactions were performed in a 96-well microplate using a BACE-1 activity detection kit (Sigma-Aldrich, MO, USA). Results were presented as units/ml (U/mL). One unit of BACE-1 activity means it hydrolyzes 1.0 pmol of 7-methoxycoumarin-4-acetyl-[Asn670, Leu671]-amyloid β/A4 precursor protein 770 fragment 667-676-(2,4-dinitrophenyl)Lys-Arg-Arg amide substrate per minute at pH 4.5, 37 °C. Data are presented as mean ± SD of three experiments.

## Conclusion

Results showed that an ethanolic extract of *D. esculentum* contained high phenolics, especially quercetin and kaempferol, that might contribute to antioxidant and anti-enzyme activities associated with AD pathogenesis in vitro. Moreover, the extract significantly decreased BACE-1 activities and Aβ42 peptide accumulation, while improving locomotor functions in *Drosophila* flies carrying human APPs and human BACE-1 or human Aβ42 in their brains. Information obtained from this study highlights that *D. esculentum* may be beneficial for the prevention or treatment of AD.

## Supplementary Information

Supplementary Figures.
