# Supplementary material for: Diplazium esculentum (Retz.) Sw. reduces BACE-1 activities and amyloid peptides accumulation in Drosophila models of Alzheimer’s disease
Source: Sci Rep. 2021 Dec 10;11:23796. doi: 10.1038/s41598-021-03142-w (PMC8664832; doi:10.1038/s41598-021-03142-w)

SUPPLEMENTARY DATA

Supplementary Figure S1:

High performance liquid chromatograms showing retention time (*Rt*) of (A.) 100 µg/mL of rutin (17.201 min), (B.) 10 mg/mL of *D. esculentum* extract, (C.) mixtures of 100 µg/mL of rutin and 10 mg/mL of *D. esculentum* extract at 280 nm.

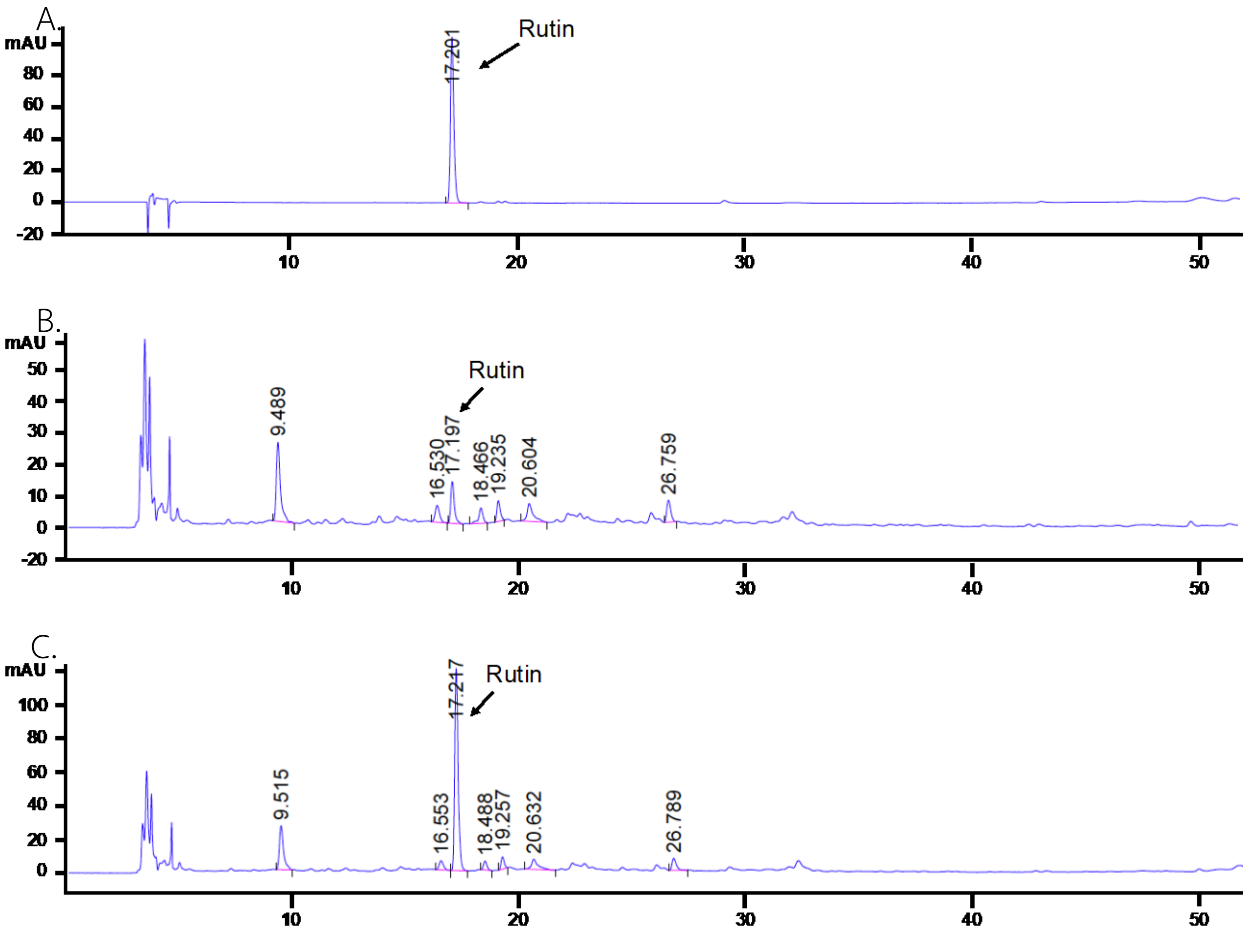

Supplementary Figure S2:

High performance liquid chromatograms showing retention time (*Rt*) of (A.) 100 µg/mL of quercetin (28.507 min), (B.) 10 mg/mL of acid hydrolyzed *D. esculentum* extract, (C.) mixtures of 100 µg/mL of quercetin and 10 mg/mL of acid hydrolyzed *D. esculentum* extract at 280 nm.

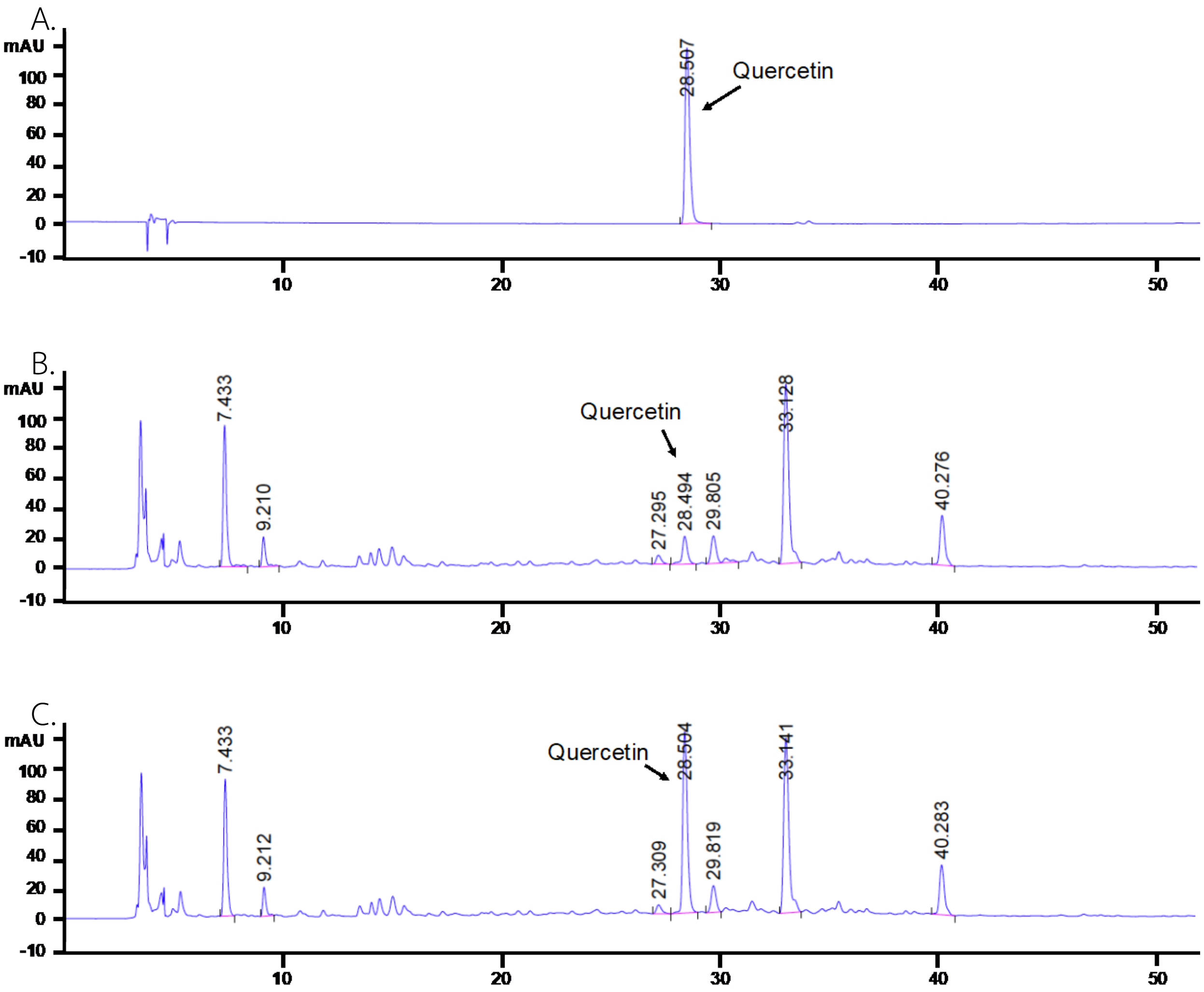

Supplementary Figure S3:

Schematic diagrams of *Drosophila* crosses and studies. (A.) a schematic diagram of *Drosophila* cross between UAS-APP-BACE-1 and elav-GAL4 to study the effect of *D. esculentum* extract on the human amyloid cascades because the F1 progenies were expressed human APP and BACE-1 in the brain region (B.) a schematic diagram of *Drosophila* cross between UAS-Aβ42 and elav-GAL4 to study the direct effect of *D. esculentum* extract on Aβ42 accumulation.

A.

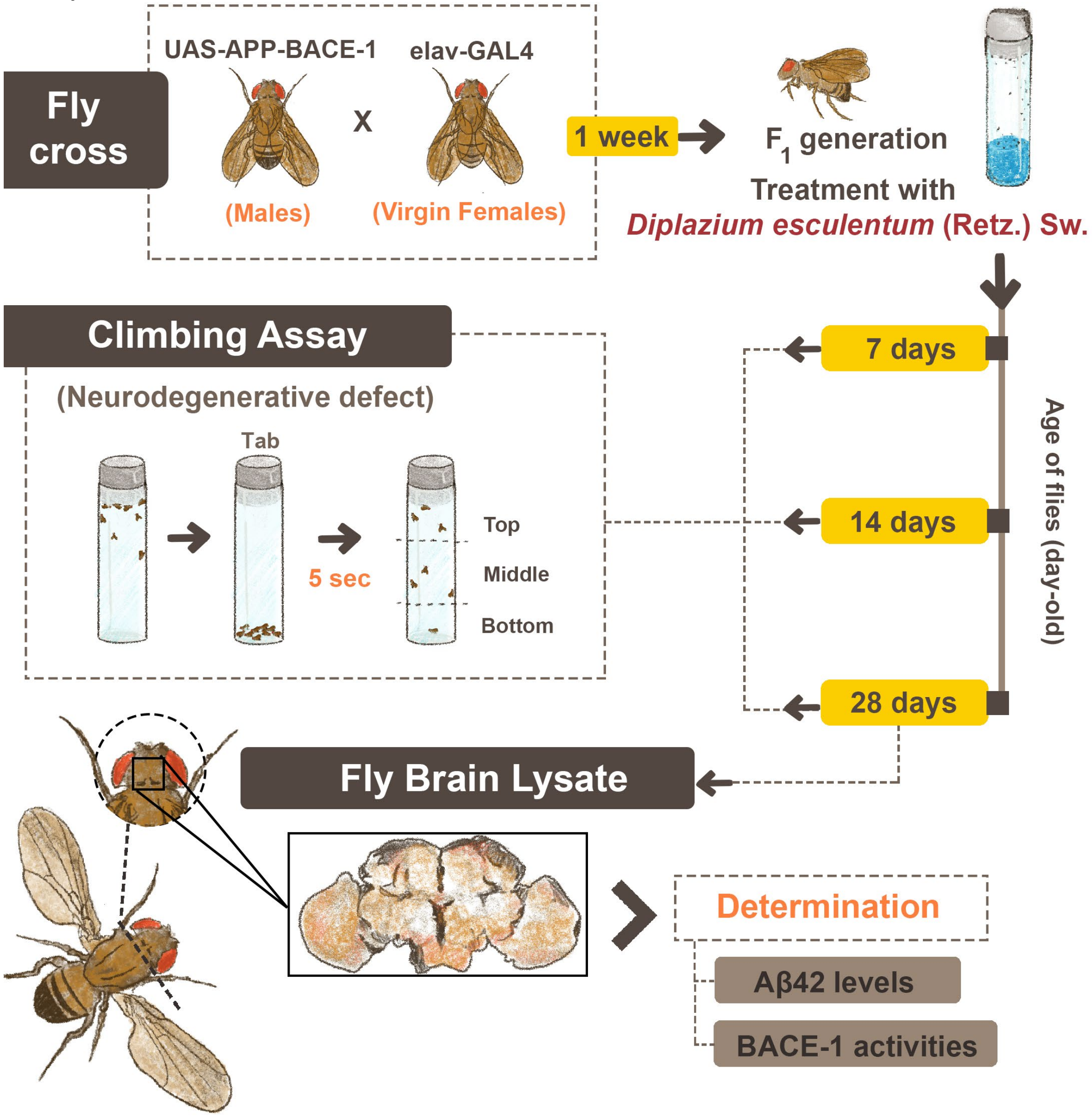

Supplementary Figure S3 (Cont.):

Schematic diagrams of *Drosophila* crosses and studies. (A.) a schematic diagram of *Drosophila* cross between UAS-APP-BACE-1 and elav-GAL4 to study the effect of *D. esculentum* extract on the human amyloid cascades because the F1 progenies were expressed human APP and BACE-1 in the brain region (B.) a schematic diagram of *Drosophila* cross between UAS-Aβ42 and elav-GAL4 to study the direct effect of *D. esculentum* extract on Aβ42 accumulation.

B.

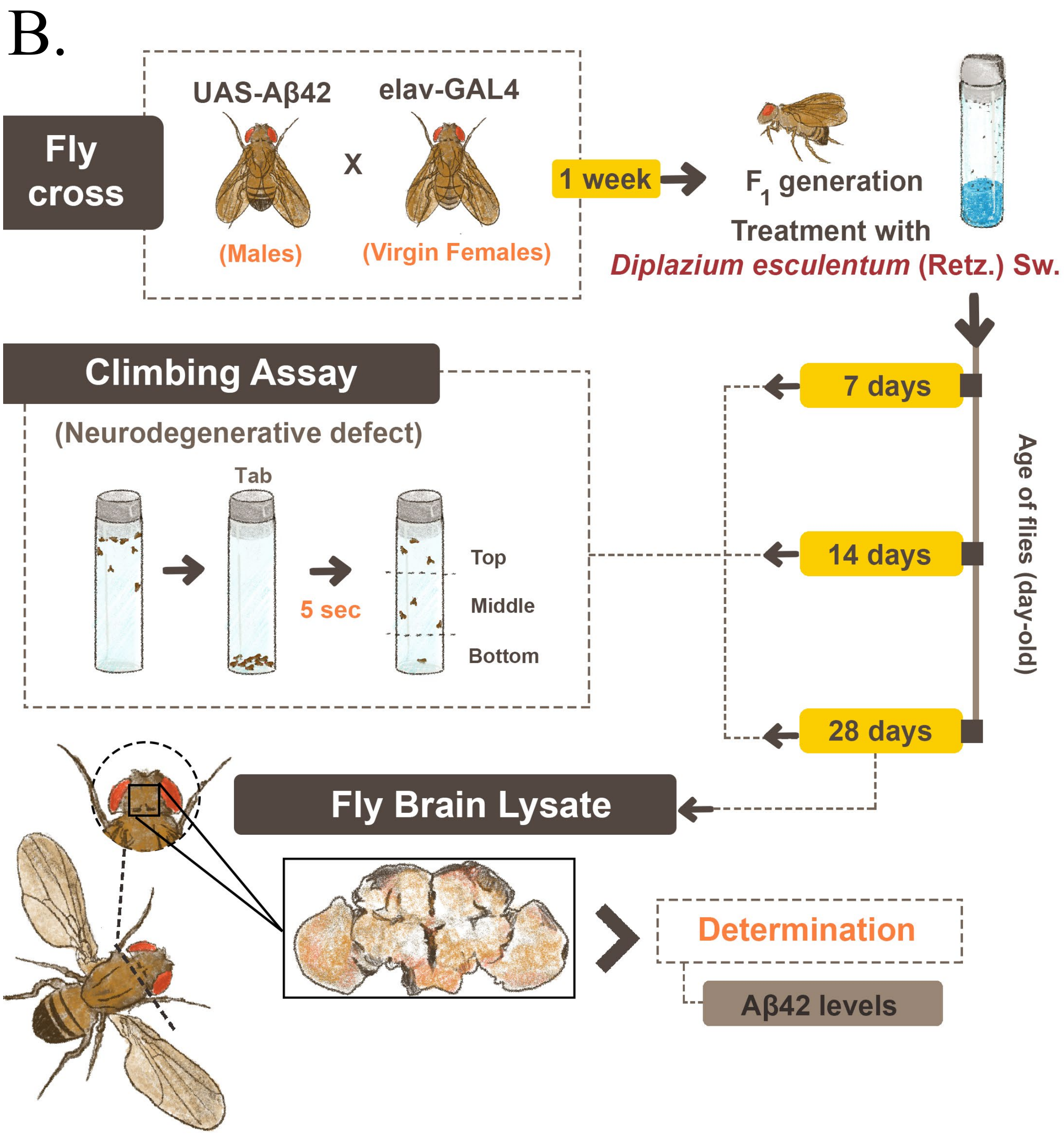

Supplement: Supplementary file 1 — Supplementary Figures. [file 41598_2021_3142_MOESM1_ESM.pdf]
